# A Finite Element Study on the Treatment of Thoracolumbar Fracture with a New Spinal Fixation System

**DOI:** 10.1155/2021/8872514

**Published:** 2021-04-10

**Authors:** Hui Guo, Jiantao Li, Yuan Gao, Shaobo Nie, Chenliang Quan, Jia Li, Wei Zhang

**Affiliations:** Department of Orthopaedics, Chinese PLA General Hospital, No. 28 Fuxing Road, Beijing 100853, China

## Abstract

**Objective:**

In this study, the mechanical properties of the new spinal fixation system (NSFS) in the treatment of thoracolumbar fractures were evaluated by the finite element analysis method, so as to provide a mechanical theoretical basis for the later biomechanical experiments and clinical experiments.

**Methods:**

T12-L2 bone model was constructed to simulate L1 vertebral fracture, and three models of internal fixation systems were established on the basis of universal spinal system (USS): Model A: posterior short-segment fixation including the fractured vertebra (PSFFV); Model B: short-segment pedicle screw fixation (SSPF); Model C: new spinal fixation system (NSFS). After assembling the internal fixation system and fracture model, the finite element analysis was carried out in the ANSYS Workbench 18.0 software, and the stress of nail rod system, fracture vertebral body stress, vertebral body mobility, and vertebral body displacement were recorded in the three models.

**Results:**

The peak values of internal fixation stress, vertebral body stress, vertebral body maximum displacement, and vertebral body maximum activity in Model C were slightly smaller than those in Model B.

**Conclusions:**

Compared with the traditional internal fixation system, the new spinal internal fixation system may have the mechanical advantage and can provide sufficient mechanical stability for thoracolumbar fractures.

## 1. Introduction

Spinal fractures account for 5%-6% of systemic fractures, and thoracolumbar fractures are the most common [1, 2]. The lumbar spine is more susceptible to the load-bearing force than the thoracic and cervical spine [3]. Stability is an important factor in the treatment of vertebral column fractures. The occurrence of burst fractures is due to axial compression and damage to the anterior and middle columns, which may lead to instability and spinal cord injury. In the past thirty years, surgical management has made tremendous progress, including various surgical stabilization strategy [4, 5]. Pedicle screw-rod system internal fixation is the most commonly used surgical method for the treatment of thoracolumbar fractures, including fracture reduction, correction of kyphosis, relief of nerve compression, and reconstruction of spinal stability. It can achieve fracture reduction, correct kyphosis, relieve nerve compression, and reconstruct spine stability [6–8]. The reduction mechanism of the pedicle screw-rod system is to restore the height of the fractured vertebral body through indirect traction, so as to correct kyphosis. The indirect reduction method cannot restore the height of fracture vertebral body and kyphotic deformity to a limited extent and cannot fully anatomize the reduction. After reduction, a cavity will be formed in the vertebral body, resulting in insufficient front support and further loss of vertebral body height in the long term. In some patients, kyphosis even worsened, and residual back pain or even failure of internal fixation occurred [9, 10].

In order to solve the problems of poor reduction of traditional instruments and insufficient anterior support, the author designed a new set of spinal internal fixation instruments, which include intravertebral distraction reduction device, minimally invasive pedicle bone graft supporting nail, vertebral implant, connector, and fixation system. In theory, this set of instruments can achieve anatomical reduction, adequate anterior support, and sufficient stability of the spine and, finally, solve the problem of loss of vertebral height in long-term fractures and avoid permanent low back pain or internal fixation failure. In order to prove the mechanical advantages of the new internal fixation system, this study compares the mechanical differences between the NSFS and the traditional internal fixation system in the treatment of thoracolumbar fractures by means of finite element analysis. The purpose of this study involved exploring the advantages and disadvantages of NSFS, PSFFV, and SSPF for the fixation of L1 vertebral fracture by using finite element analysis. The report is as follows.

## 2. Methods

### 2.1. Establishment of Finite Element Model

**Figure 1:**
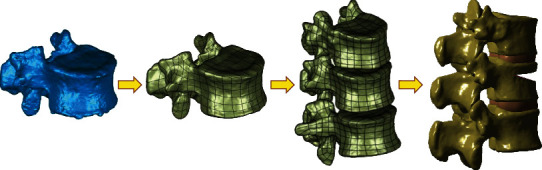
Establishment of vertebral body model.

The CT images of thoracolumbar vertebrae were imported into the three-dimensional reconstruction software mimics 20 (Materialise Company, Belgium) in DICOM format, and the three vertebral models of T12 L1 and L2 were reconstructed. Import the STL file format into Geomagic 2012 (Geomagic, USA) to build a curved surface model and perform V-shaped osteotomy on L1 vertebrae to construct a fracture model (Figure 1).

**Figure 2:**
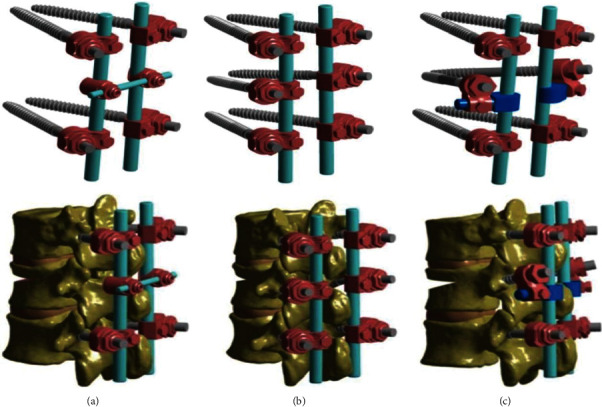
Three dimensional solid modeling: (a) USS four nails+2 rods+1 transverse joint (crosslink); (b) USS six nails+2 rods; (c) NSFS: USS four nails+2 rods+two new Schanz nails+connectors.

Using SolidWorks 2015 (Dassault, France) establishes a three-dimensional model of pedicle screw rod system and assembles the pedicle screw rod system and vertebral model. Three fixation models and postassembly models of pedicle screw rod system is constructed, as shown in Figure 2.

The bone and the intervertebral disc meshed into eight-node hexahedral elements with Solid 187 with a mesh size of 1 mm were chosen. There are 436283 and 711037 units in Model A, 459409 nodes and 750985 units in Model B, and 493072 nodes and 809052 cell units in Model C. The assembled entity model is imported into the software ANSYS Workbench 18.0 (ANSYS company, USA) for Boolean operation analysis. Units and nodes of model were shown in Table 1.

### 2.2. Material Properties, Boundary Conditions, and Loading

**Figure 3:**
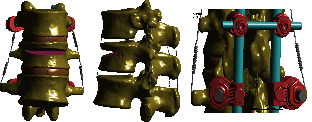
The ligaments were established according to the physiological characteristics.

According to the attachment positions, the spinal ligaments were established (Figure 3) and defined as nonlinear hyperplastic material using Combine 39, which only allowed for tension deformation without compression behavior. The mechanical behaviors of the spinal ligaments were based on nonlinear stress-strain curves [11]. The nonlinear properties of ligaments were shown in Table 2. The elastic properties and Poisson's ratio of various structural materials were shown in Table 3 [11, 12].

The contact relationship was set as follows. The interfaces between the screw and bone were set to be bonded. The cortical bone and cancellous bone were set to be bonded. The vertebral body was bound to the adjacent intervertebral disc. And nucleus pulposus and annulus fibrosus were set to be bonded.

In order to simulate the real stress of human lumbar vertebrae, the lower endplate of L2 was restrained and fixed. 500 N axial compression force was applied to the upper endplate of T12 of three groups of models, and pure bending moment of 7.5 Nm was applied to all directions at the same time.

### 2.3. Evaluation Index

The evaluation indexes include values and distribution of internal fixation stress of three finite element models under flexion, extension, right flexion, and right rotation loading conditions; fracture vertebral body stress; vertebral range of motion (ROM); and vertebral displacement.

## 3. Results

### 3.1. Stress Comparison of Internal Fixation System

**Figure 4:**
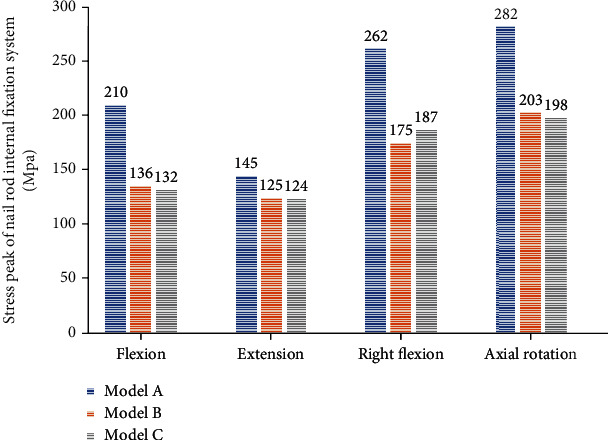
The histogram peak value of internal fixation stress in nail rod system.

The peak value of stress shows that Model A has the largest peak value of stress, which is 210 MPa for forward bending, 145 MPa for backward extension, 262 MPa for right flexion, and 282 MPa for right rotation. The comparison results of the peak values of stress in three Models are A > B > C for forward bending, backward extension, and right rotation and A > C > B for right flexion (Figure 4).

**Figure 5:**
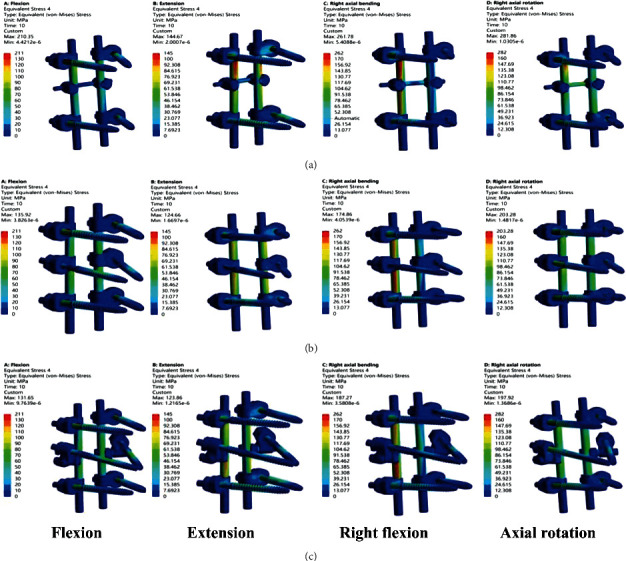
Distribution of the maximum Von Mises stresses in the nail-rod system.

The stress distribution nephogram shows that the stress distribution of the nail bar in Model A is more concentrated, mainly on the root of the screw and the rod above the cross-link; there is no obvious stress concentration in Model B and C. Stress distribution is more scattered, the two screws added in the middle share part of the stress, and the stress on the rod is evenly distributed on the whole (Figure 5).

### 3.2. Comparison of Fracture Vertebral Body Stress

**Figure 6:**
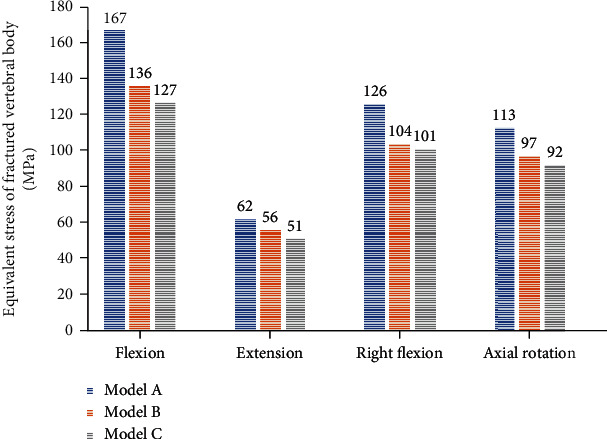
Histogram of peak stress of fractured vertebral body.

Comparing the peak stress of the fractured vertebral body, the peak stress of Model A is the largest, which is 167 MPa of flexion, 62 MPa of posterior extension, 126 MPa of right flexion, and 113 MPa of right rotation. The comparison results of the peak stress of the three groups are flexion, extension, right flexion, and right rotation under all four working conditions: A > B > C (Figure 6).

**Figure 7:**
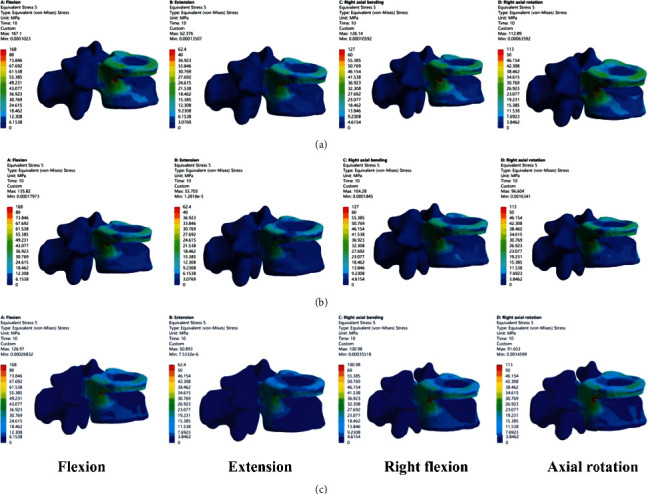
Distribution of the maximum Von Mises stresses in fractured vertebral body.

The stress distribution nephogram of vertebral body shows that the stress is mainly concentrated on the upper endplate and the middle column, especially the apex of “V”-shaped osteotomy. Compared with the stress distribution transmitted to the lower end plate, the stress distribution of the lower end plate in Model A was the most scattered, which may be related to the screw support in the fracture plane of Model B and C (Figure 7).

### 3.3. Comparison of Vertebral Displacement and Mobility

Vertebral displacement showed that Model A had the largest vertebral displacement, which is 2.1 mm for forward flexion, 0.8 mm for posterior extension, 1.9 mm for right flexion, and 1.8 mm for right circumflexion. The comparison results of vertebral displacement of the three Models were flexion forward and right flexion A > B = C; the posterior extension and right rotation A > B > C.

**Figure 8:**
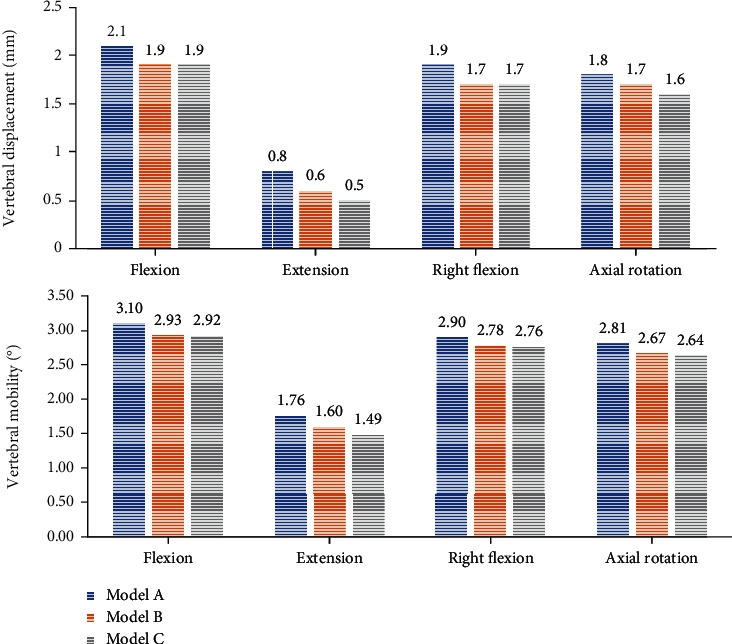
Histogram of displacement and mobility of vertebral body.

The ROM of vertebral body in Model A was 3.10° in flexion, 1.76° in extension, 2.90° in right flexion, and 2.81° in right rotation. The comparison of ROM among the three Models was as follows: flexion, extension, right flexion, and right rotation A > B > C. (Figure 8).

## 4. Discussion

Thoracolumbar fractures are often treated with posterior short segment fixation [13–15]. There are two traditional short segment fixation methods: posterior short-segment fixation including the fractured vertebra (PSFFV) and short-segment pedicle screw fixation (SSPF) [16]. In this study, by means of finite element simulation mechanical analysis, we compared the mechanical differences among PSFFV (Model A), traditional SSPF (Model B), and NSFS (Model C). The results showed that the NSFS had significant mechanical advantages in the fixation of vertebral fractures.

Comparison between Model C and Model A, Internal fixation stress: the stress distribution of the internal fixation system in Model C is more scattered, and the peak value of stress is smaller than that in Model A, which indicates that the fracture risk of the internal fixation system in Model C is lower than that in Model A. Spine stability: the vertebral body stress, vertebral displacement, and ROM of the internal fixation system in Model C were lower than those in Model A, which proved that the spinal stability of the internal fixation system in Model C was stronger. Through the study and comparison, we think that the NSFS has obvious mechanical advantages over the PSFFV, mainly because the new internal fixation system increases the plane nail placement of the injury vertebra, which makes the spine more stable, the stress more scattered, and reduces the risk of breaking the nail and the rod. This is consistent with the research results of Liao et al. [17], who found that for vertebral fracture, the fixation method of SSPF has more stable fixation strength and more scattered stress than that of PSFFV. In a biomechanical study of fresh frozen cadaver, Baaj et al. [18] found that compared with fixation across injured vertebrae, fixation of injured vertebrae can increase structural stability by 25%, and also has the quadrilateral effect and suspension effect to avoid the fixation of cross segments. It has the mechanical advantages of dispersing and reducing the internal fixation stress and significantly reducing the failure of internal fixation. Elbehairy et al. [19] found that 32 patients with thoracolumbar fractures fixed by SSPF can provide more stable vertebral fixation strength.

Comparison between Model C and Model B, Internal fixation stress: the distribution of internal fixation stress in the two models is relatively similar, which is relatively scattered. Except that the stress peak value of Model C was greater than that of Model B under the right flexion condition. The stress peak value of Model C was lower than that of Model B under other conditions, indicating that the fracture risk of the NSFS of Model C was lower. Spine stability: the vertebral body displacement and vertebral body ROM of the internal fixation system in Model Care were less than that in Model B, which proves that the spinal stability in Model C is stronger than that in Model B. Through research and comparison, we believe that the NSFS has significant mechanical advantages over the traditional SSPF, which may be related to the way the screw is placed in the injured vertebral plane. The two screws enter into the vertebral body through the middle point of the outer wall of the pedicle root, respectively, so as to provide more cortical bone fixation, increase the cohesion angle of the screw placement, make the screw placement longer, and increase the stability of the spine. Dvorak et al. [20] compared pedicle internal fixation with pedicle external fixation, proving that pedicle external fixation can provide better spinal stability; Klane et al. [21] proved that there was little difference in biomechanics between pedicle internal screw and pedicle external screw through biomechanical experiments, through the study of 8 fresh cadavers. Barber et al. [22] found that the greater the cohesion angle of pedicle screws, the more resistant it was to pull out stress and provided better spinal stability. In addition, we found that the NSFS is not collinear (the middle injuried vertebra is outside), which provides multiplane fixation control, thus increasing its stability against loads in different directions.

Besides, the research shows that there are some shortcomings in the traditional fixation system: (1) insufficient fracture reduction and (2) insufficient anterior support [23–25]. (3) All lumbar paraspinal muscles were rendered useless, which may be another major limitation of this study. The traditional SSPF system replaces the fractured vertebral body by indirect traction. The fractured vertebral body can be effectively reduced by the traction of the fibrous annulus of the intervertebral disc, but the central bone mass of the anterior and middle column cannot be pulled, resulting in a central collapse defect, resulting in insufficient reduction [26, 27]. Although the height and shape of the fractured vertebral body are restored after indirect reduction, it is difficult to restore the trabecular structure in the vertebral body. The traditional fixation method of injured vertebrae is to place nails before reduction, which occupies the channels of reduction and bone grafting in the vertebral body, thus cannot be further operated in the injured vertebrae, resulting in a cavity in the vertebral body and insufficient anterior support [28]. The NSFS designed in this study can solve the above problems, and its principle is as follows: first, it is the same as the internal fixation system of cross injured vertebrae, and it is indirectly braced and fixed with cross injured vertebrae nails; then, through the pedicle passage at the midpoint of the transverse process root of injured vertebra, a new type of intravertebral distraction device was used for direct distraction reduction in the vertebral body and anatomical reduction of the fractured vertebral body. Then, the vertebral cavity was filled with bone graft to provide anterior support of vertebral body, and finally, the nails were placed on both sides. After connecting the rods, NSFS were combined into a whole. It realizes the anatomical reduction of the fractured vertebral body, provides sufficient anterior support of injured vertebra, and ensures the stability of the spine at the same time.

This study is also limited by this study is only a finite element analysis. Stress and displacement of the spine were investigated as there are considered important varies. The biomechanical propriety was well described in this study but not all biomechanical parameters. The further biomechanical experiments need to be performed to prove the results of this study.

## 5. Conclusion

The results of finite element mechanical analysis show that the new spinal internal fixation system may have mechanical advantages over the traditional internal fixation system in the treatment of vertebral fracture, which can provide sufficient mechanical stability at the fracture end, and it is worth further promotion and application.

## Figures and Tables

**Table 1:** Unites and nodes of model.

Model	Unites	Nodes
A	436283	711037
B	459409	750985
C	493072	809052

**Table 2:** Parameters of force-deflection-curve of the ligaments.

ALL		PLL		FL		ISL		SSL		ITL		CL	
D (mm)	F (N)	D (mm)	F (N)	D (mm)	F (N)	D (mm)	F (N)	D (mm)	F (N)	D (mm)	F (N)	D (mm)	F (N)
-50	0	-50	0	-50	0	-50	0	-50	0	-50	0	-50	0
0	0	0	0	0	0	0	0	0	0	0	0	0	0
0.23173	11.501	0.93583	6.0533	3.6419	4.8781	1.2952	4.2683	1.981	4.8781	3.779	6.0976	1.8984	6.0533
0.40107	22.397	1.025	12.107	4.3886	23.171	1.6762	14.024	2.4838	20.732	4.9676	10.976	2.4153	13.317
0.5615	43.584	1.1587	32.082	4.7848	40.854	1.8743	23.171	2.88	39.634	5.5619	22.561	3.057	39.952
0.67736	65.375	1.2567	55.085	5.0743	62.805	2.0267	41.463	3.1543	59.756	6.0952	42.683	3.2799	61.138
0.81105	106.54	1.4617	148.31	5.379	107.32	2.1638	61.585	3.6419	103.05	6.5524	64.634	3.6096	105.93
1.016	200.97	1.5865	248.79	5.6076	151.22	2.3771	104.88	4.0076	151.22	7.0705	99.39	3.877	151.33
1.0695	249.4			5.8971	249.39	2.5448	158.54	4.2971	204.88	7.5886	146.95	4.0374	202.79
						2.6514	209.15	4.5105	249.39	7.9238	200	4.1533	249.4
						2.6971	249.39			8.1676	248.78		

ALL: anterior longitudinal ligament; PLL: posterior longitudinal ligament; FL: flaval ligament; ISL: interspinous ligament; SSL: supraspinous ligament; ITL: intertransverse ligament; CL: capsular ligaments; D: deflection; F: force.

**Table 3:** The material properties used in the finite-element model.

Material	Young modulus E (MPa)	V Poisson ratio
Cortical bone/endplate	12000	0.3
Cancellous bone	100	0.2
Posterior arch	3500	0.25
Annulus fibrosus	4.2	0.4
Nucleus pulposus	1	0.49
Spinal instrumentation	110000	0.3

## Data Availability

Data are available from the corresponding author upon request.

## Abbreviations

PSFFV: Posterior short-segment fixation including the fractured vertebra
SSPF: Short-segment pedicle screw fixation
NSFS: New spinal fixation system
USS: Universal spinal system
USA: United States of America
ROM: Range of motion.
